# Efficacy and Mechanism of the Action of Live and Heat-Killed *Bacillus coagulans* BC198 as Potential Probiotic in Ameliorating Dextran Sulfate Sodium-Induced Colitis in Mice

**DOI:** 10.1021/acsomega.3c07529

**Published:** 2024-02-20

**Authors:** Yen-Chun Koh, Ya-Chu Chang, Wei-Sheng Lin, Siu-Yi Leung, Wei-Jen Chen, Shiuan-Huei Wu, Yu-Shan Wei, Chiau-Ling Gung, Ya-Chun Chou, Min-Hsiung Pan

**Affiliations:** † Institute of Food Sciences and Technology, National Taiwan University, Taipei 10617, Taiwan; ‡ Department of Food Science, National Quemoy University, Quemoy 892, Taiwan; § Biotech Department, Syngen Biotech Co., Ltd., Tainan 744094, Taiwan; ∥ Research and Development Department, Syngen Biotech Co., Ltd., Tainan 744094, Taiwan; ⊥ Department of Medical Research, China Medical University Hospital, China Medical University, Taichung City 40402, Taiwan; # Department of Health and Nutrition Biotechnology, Asia University, Taichung City 41354, Taiwan

## Abstract

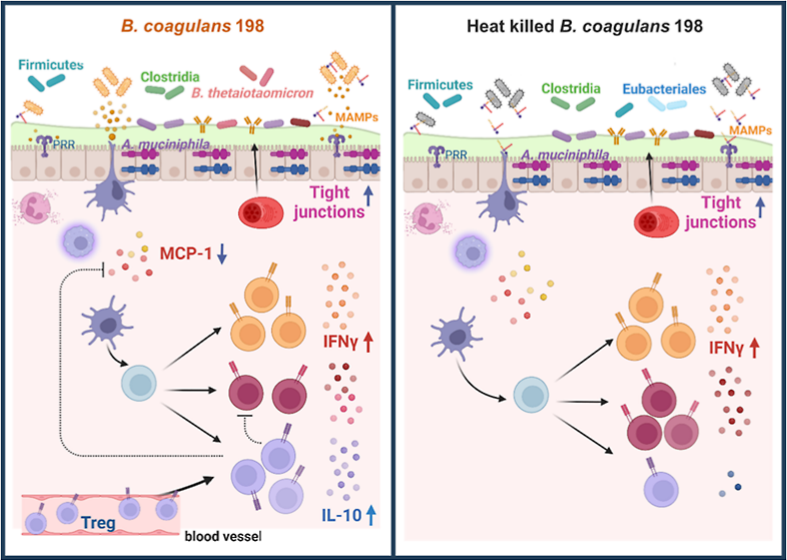

Inflammatory bowel disease alters the gut microbiota, causes defects in mucosal barrier function, and leads to dysregulation of the immune response to microbial stimulation. This study investigated and compared the efficacy of a candidate probiotic strain, *Bacillus coagulans* BC198, and its heat-killed form in treating dextran sulfate sodium-induced colitis. Both live and heat-killed *B. coagulans* BC198 increased gut barrier-associated protein expression, reduced neutrophil and M1 macrophage infiltration of colon tissue, and corrected gut microbial dysbiosis induced by colitis. However, only live *B. coagulans* BC198 could alleviate the general symptoms of colitis, prevent colon shortening, and suppress inflammation and tissue damage. At the molecular level, live *B. coagulans* BC198 was able to inhibit Th17 cells while promoting Treg cells in mice with colitis, reduce pro-inflammatory MCP-1 production, and increase anti-inflammatory IL-10 expression in the colonic mucosa. The live form of *B. coagulans* BC198 functioned more effectively than the heat-killed form in ameliorating colitis by enhancing the anti-inflammatory response and promoting Treg cell accumulation in the colon.

## Introduction

Inflammatory bowel disease (IBD) is a chronic inflammation of the gut caused by genetic variation, adverse environmental factors, dysbiosis of the gut microbiota, and dysregulated immune responses.^1^ Before IBD develops, dysfunction of intestinal mucosal immunity occurs, including impairment of the gut barrier function and overactivation of the pro-inflammatory immune response, leading to chronic inflammation of the intestine.^2^

Goblet cells and MUC2 protein production are reduced in IBD patients^3−5^ and the apical junctional complex between adjacent intestinal epithelial cells is dissociated,^6^ leading to impaired gut barrier function and increased intestinal permeability.^6^ After the gut barrier is impaired, a large number of bacteria in the gut lumen penetrate the intestinal mucosal layer, triggering the polarization of macrophages into the pro-inflammatory M1 phenotype and the secretion of chemokines that cause large numbers of neutrophils to infiltrate the intestinal mucosa and submucosa, resulting in the accumulation of many neutrophils and M1 macrophages in intestinal tissue. The free radicals generated by these innate immune cells are the main factor causing damage to intestinal tissue.^7^

In the adaptive immune response of patients with IBD, pro-inflammatory effector T cells are increased and anti-inflammatory regulatory T cells are decreased, thereby driving chronic inflammation of the intestine.^1^ Among T cells, the ratio of pro-inflammatory Th17 cells to anti-inflammatory Treg cells is generally dynamically balanced through regulation by gut microbes and cytokines;^8^ however, in chronic inflammation, pro-inflammatory cytokines affect the conversion of Treg cells to Th17 cells, resulting in an imbalance in the Th17/Treg cell ratio, leading to continual inflammation.^9^ Britton et al. found that the microbiota of humans with IBD altered the balance of gut Th17 and Treg cells and exacerbated colitis in mice,^10^ suggesting that the dysbiosis of the gut microbiota of IBD patients may be responsible for the imbalance in the T cell immune response.

Probiotic strains of *Bacillus coagulans* are used in the prevention and treatment of gastrointestinal disorders, including irritable bowel syndrome and antibiotic-associated diarrhea,^11^ and have been shown to significantly improve IBD.^12−15^ Moreover, the spore-forming property of *B. coagulans* increases its rate of survival after gastrointestinal digestion, making it an ideal species for gastrointestinal disorders. However, few studies have focused on the effects of heat-killed *B. coagulans* on IBD; therefore, further research is necessary in this area.

Probiotics, such as dead bacterial bodies or bacterial fragments, which retain their critical probiotic properties after heat treatment are known as heat-killed probiotics.^16^ After heat treatment, the effector molecules embedded in the cell walls of probiotics may be released from the fragmented bacterial bodies. These effector molecules, because of their smaller size, have a better chance than intact probiotics of passing through the intestinal mucus layer and interacting with the host’s intestinal epithelial cells and dendritic cells;^16−18^ therefore, heat-killed probiotics may be better able to improve immunomodulatory effects than their live forms. On the other hand, probiotics may also lose their activity and fail to produce beneficial enzymes or metabolites after heat treatment, thus showing impaired probiotic properties.^19^ Therefore, whether the heat-killed form of a probiotic strain has enhanced physiological efficacy compared to its live form must be tested experimentally.

Therefore, the present study aimed to investigate whether the live *B. coagulans* BC198 strain can better ameliorate colitis in mice by immunomodulation than its heat-killed form. Intestinal mucosal immunity, including gut barrier function, innate and adaptive immune responses, and gut microbiota, was determined to compare different effects between live and heat-killed *B. coagulans* BC198.

## Results

### Live *B. coagulans* BC198 Prevented Colon Shortening Caused by Colitis in Mice

**Figure 1:**
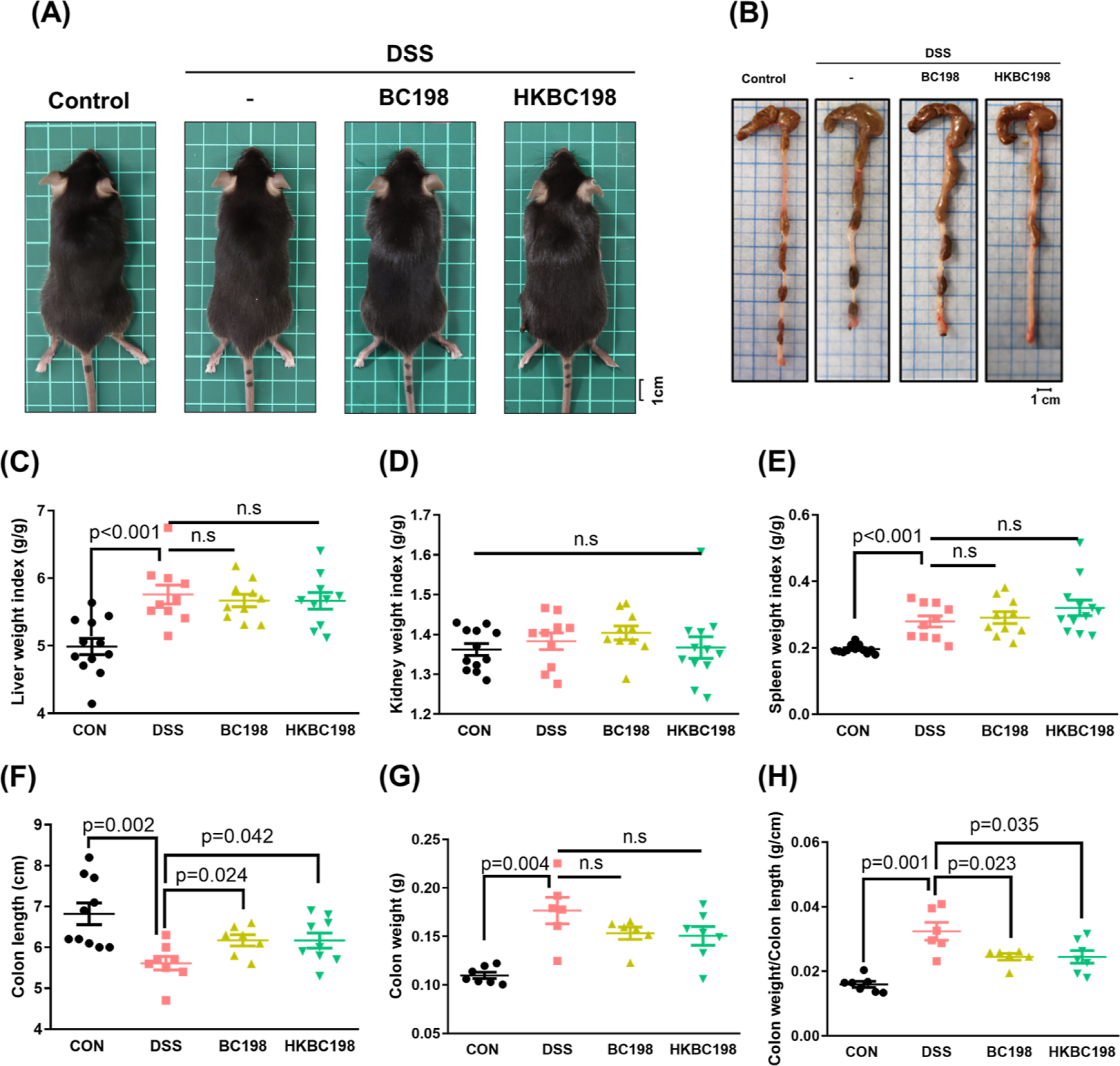
Effects of live and heat-killed *B. coagulans* BC198 supplementation on DSS-induced colitis in mice. The appearance of (A) mice and (B) colon of mice treated with PBS (control group), DSS (DSS group), DSS and live *B. coagulans* BC198 (BC198 group), and DSS and heat-killed *B. coagulans* BC198 (HKBC198 group); (C) liver weight index; (D) kidney weight index; (E) spleen weight index; (F) colon length; (G) colon weight; and (H) colon weight/colon length ratio. Data are shown as the mean ± standard error. The groups were compared using two-tailed Student’s *t*-tests, with *p* < 0.05 indicating a significant difference.

Mice consuming dextran sulfate sodium (DSS)-containing water show symptoms of weight loss, diarrhea, and rectal bleeding similar to those of human ulcerative colitis.^20^ The severity of these three symptoms is scored to determine the disease activity index (DAI), which is used to monitor colitis progression and severity.^21^ In this study, there were no obvious differences in appearance, but significant differences in liver and spleen weights between the control and DSS groups (*p* < 0.001; Figure 1A), and supplementation with live (BC198 group) or heat-killed (HKBC198 group) *B. coagulans* BC198 did not affect organ weights compared to the DSS group (Figure 1C–E). DSS consumption led to colon shortening compared to the control group (Figure 1F), and both supplementations showed ameliorative effects on colon length (Figure 1B,F) and the ratio of colon weight/colon length (Figure 1H).

### Live *B. coagulans* BC198 Reduced the DAI of Mouse Colitis

**Figure 2:**
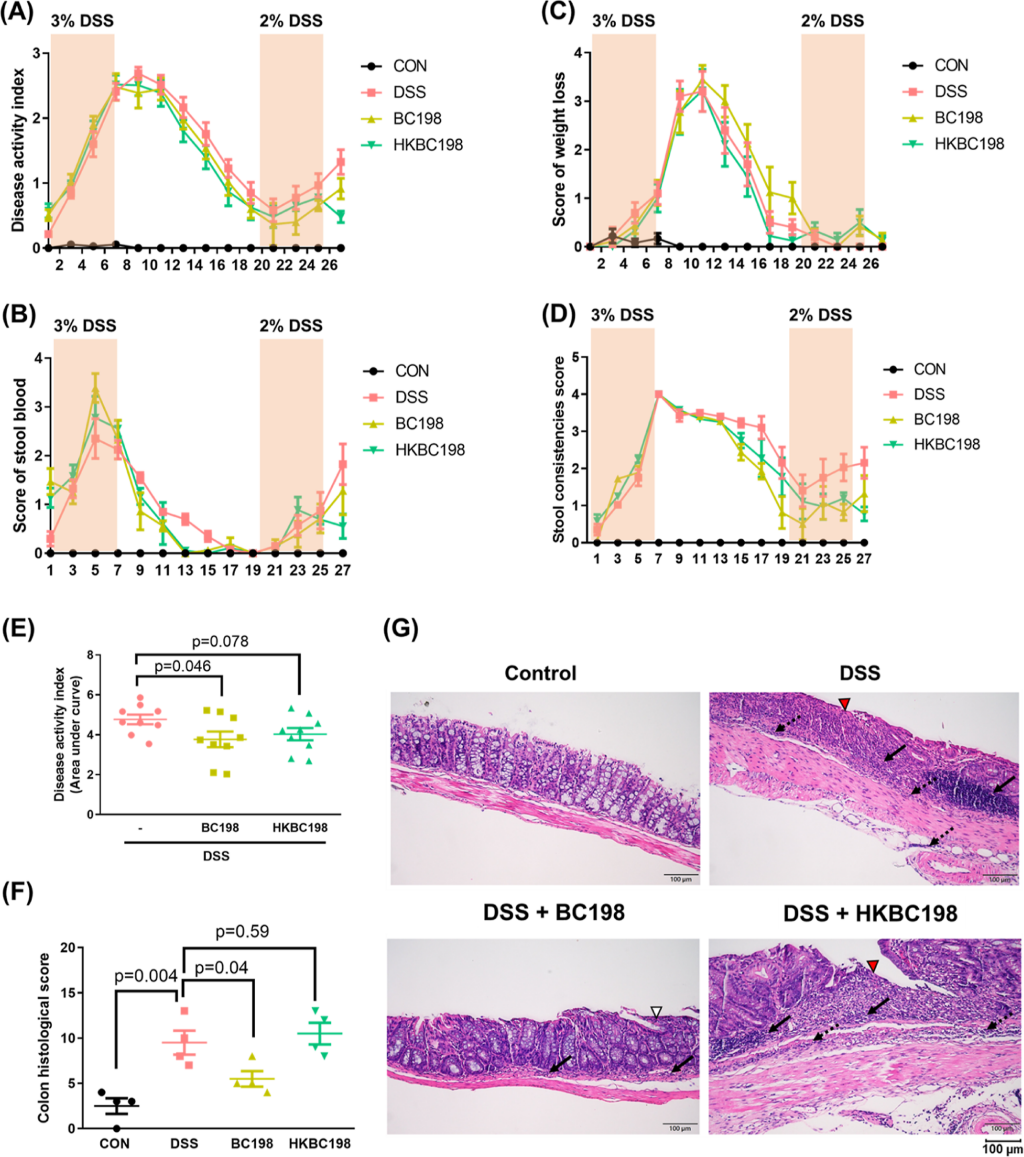
Live *B. coagulans* BC198 ameliorates colitis and suppresses colon inflammation. (A) DAI; (B) stool blood score; (C) average body weight loss score; and (D) stool consistency score of each group. (E) The AUC of DAI; (F) histological score evaluation of the colon; and (G) representative image of paraffin-embedded distal segments of colon tissue stained with hematoxylin and eosin (H&E) (200× magnification; scale bar = 100 μm). Arrows: inflammatory immune cell infiltration of the mucosa (solid) and submucosa (dotted); arrowhead: ulceration (red), erosion (white). Data are shown as mean ± standard error. Groups were compared using two-tailed Student’s *t*-tests, with *p* < 0.05 indicating a significant difference.

The DAI of the DSS group increased with the number of days of DSS administration during the first and second periods of colitis induction. In comparison, the DAI of mice supplemented with live or heat-killed *B. coagulans* BC198 tended to decline during the recovery period of the first colitis cycle and the induction and recovery period of the second colitis cycle, suggesting that live and heat-killed *B. coagulans* BC198 had an alleviative effect during the development of colitis and also reduced the severity of the recurrence of colitis. The area under the curve (AUC) of DAI was calculated to quantify the effectiveness of the *B. coagulans* treatments in reducing DAI throughout the experimental period. The AUC of the BC198 group showed a significant 21% decrease compared to that of the DSS group (*p* < 0.05; Figure 2E), indicating that live *B. coagulans* BC198 had a better protective effect against the symptoms of colitis than its heat-killed counterpart (Figure 2E).

DSS-induced colonic inflammation was reflected in the shortening of the colon^20^ and histological changes in colon tissue. The colon of the DSS group was significantly shorter than that of the control group (*p* < 0.01) (Figure 1F), and the results of histological evaluation demonstrated that the DSS group had severe colon tissue inflammation (histological score of 9 points) (Figure 2F). Supplementation with live *B. coagulans* BC198 had a significant preventative effect on colon shortening (*p* < 0.05) (Figure 1F) and also caused a significant reduction in histological colon tissue inflammation (histological score of 5.5 points) (*p* < 0.05) (Figure 2F). In contrast, heat-killed *B. coagulans* BC198 did not have protective effects on the colon morphology and histology. Histological evaluation and hematoxylin and eosin staining of the colon tissue of the DSS and HKBC198 groups showed severe immune cell infiltration extending to the mucosa and submucosa and large ulcerations, crypt loss, and goblet cell depletion in the tissue structure (Figure 2G). However, in the BC198 group, immune cell infiltration was only limited to the mucosa, and the epithelium and crypt structure were intact, with only slight and localized erosion (Figure 2G).

### Live and Heat-Killed *B. coagulans* BC198 Reduced Pro-Inflammatory Innate Immune Cell Infiltration of Colon Tissue

**Figure 3:**
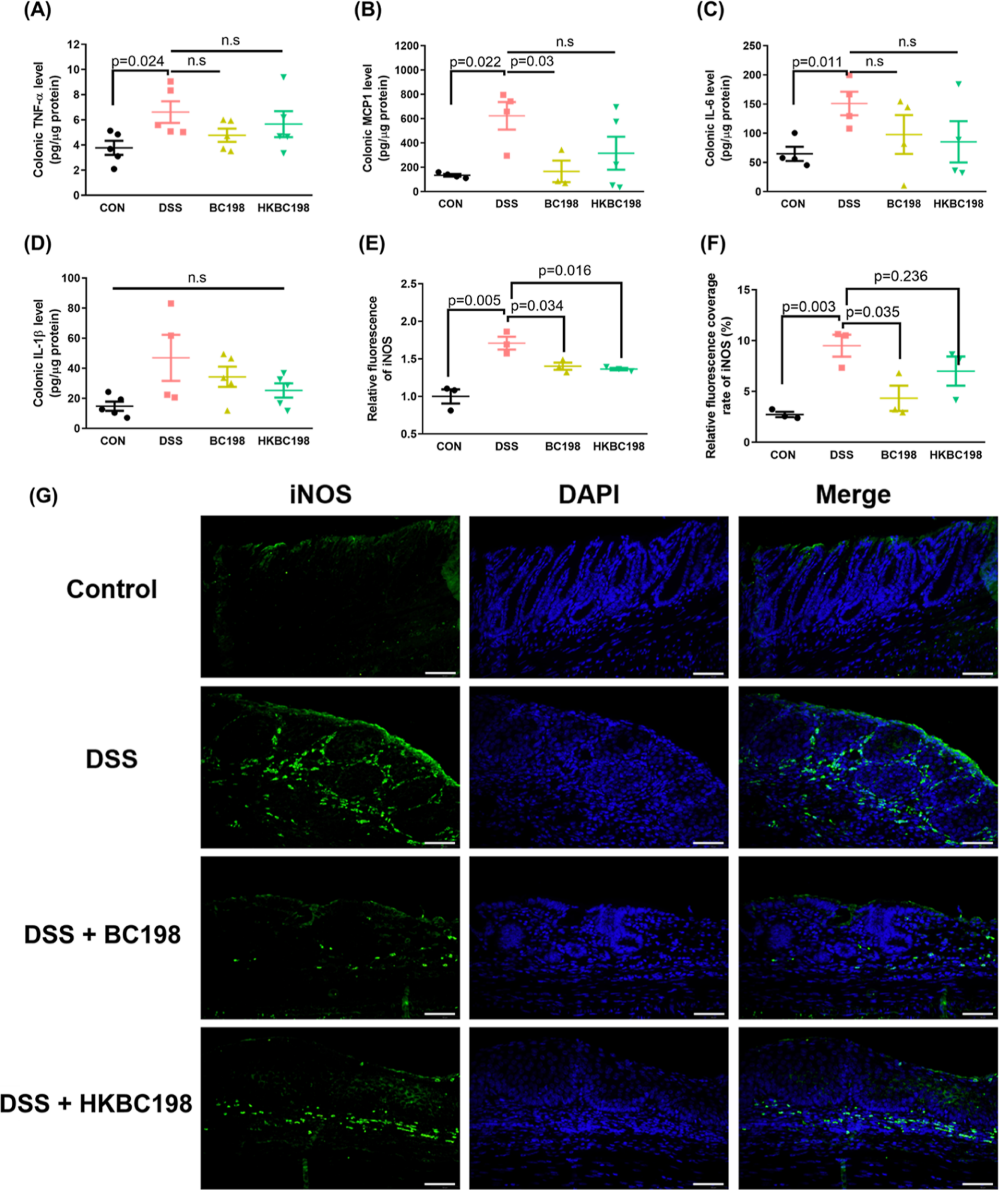
Live and heat-killed *B. coagulans* BC198 reduced pro-inflammatory innate immune cells in colon tissue. Pro-inflammatory cytokines and a chemokine in the supernatant of colonic biopsies cultured ex vivo detected by ELISA analysis. (A) TNF-α; (B) MCP-1; (C) IL-6; and (D) IL-1β. Mean (E) fluorescence intensity and (F) proportion of iNOS expressed in (G) immunofluorescence staining of iNOS protein in distal segments of colon tissue colon tissue (coverage rate) per high-power field (400× magnification) were analyzed using ImageJ. (400× magnification; scale bar = 50 μm). Data are shown as mean ± standard error. Groups were compared using two-tailed Student’s *t*-tests, with *p* < 0.05 indicating a significant difference.

To further evaluate the degree of inflammation of colon tissue, the levels of the pro-inflammatory factors TNF-α, IL-1β, IL-6, and MCP-1 were measured. TNF-α promotes inflammation and triggers apoptosis and basement membrane degradation of epithelial cells,^2^ while IL-1β, IL-6, and MCP-1 activate immune cells, promote their growth, and trigger their infiltration.^22,23^ The concentrations of IL-6, TNF-α, and MCP-1 were significantly higher in the DSS group than in the control group (*p* < 0.05) (Figure 3A–C), and the IL-1β (Figure 3D) concentration also tended to be higher, indicating significant inflammatory responses in the colon of the DSS group. In comparison, the MCP-1 concentration was significantly lower in the BC198 group than in the DSS group (*p* < 0.05), and the concentrations of IL-6 and TNF-α also tended to be lower, while there were no significant differences in the concentrations of any pro-inflammatory cytokines between the HKBC198 group and the DSS group. The DAI scores and degree of colon inflammation showed that live *B. coagulans* BC198 was more effective than the heat-killed form in improving colitis. To understand the reasons for the differences between the two groups, we conducted further experiments on the mechanism of action of live and heat-killed *B. coagulans* BC198 in ameliorating colitis.

In the case of the M1 macrophage (Figure 3E–G), immunofluorescence staining showed that iNOS, a biomarker of the macrophage, was expressed at a high level in the colon tissue of the DSS group, with the mean fluorescence intensity and tissue coverage rate of iNOS being significantly higher than those of the control group (*p* < 0.005). In comparison, the rate of iNOS coverage of the BC198 group was 54% lower than that of the DSS group (*p* < 0.05); the iNOS coverage rate of the HKBC198 group was 26% lower than that of the DSS group. These results indicate that both live and heat-killed *B. coagulans* BC198 could reduce innate immune cell infiltration, which causes tissue damage.

### Live and Heat-Killed *B. coagulans* BC198 Enhanced the Gut Barrier Function

**Figure 4:**
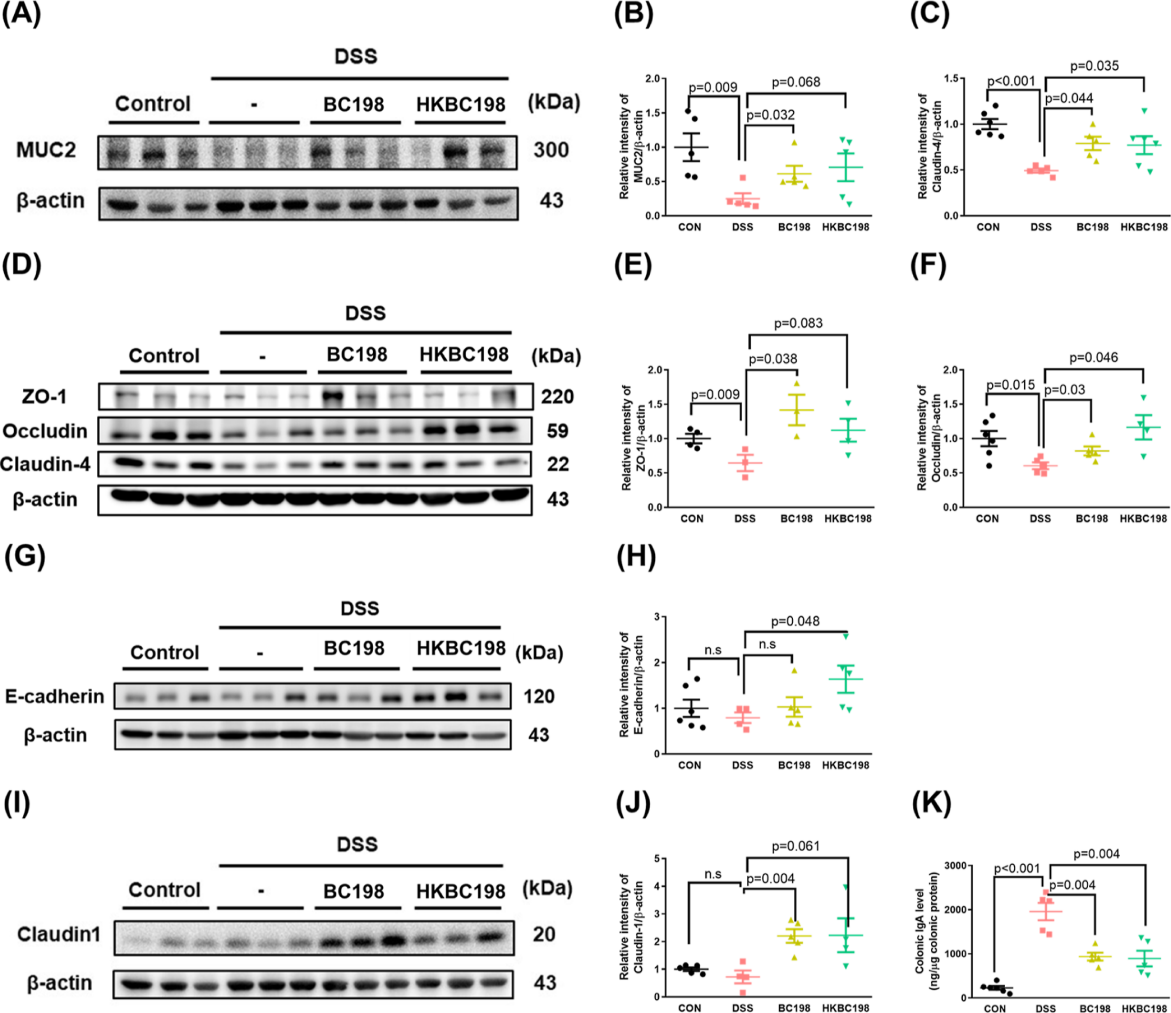
Live and heat-killed *B. coagulans* BC198 enhanced the gut barrier function. Western blot detection and quantification of (A,B) MUC2; (C–F) ZO-1, occludin, and claudin-4; (G,H) E-cadherin; and (I,J) claudin1 protein expression in colon tissue. (K) ELISA analysis of IgA in colon tissue. Data are shown as mean ± standard error. Groups were compared using two-tailed Student’s *t*-tests, with *p* < 0.05 indicating a significant difference.

The gut barrier consists of the mucus layer, epithelial layer, and immunoglobulins, which are the first defenses against the invasion of microbes from the gut lumen into the mucosa; thus, they may prevent uncontrolled immune activation.^24^ The expression of MUC2, the main protein in mucus, and junction proteins ZO-1, occludin, claudin-4, and E-cadherin is significantly reduced in IBD patients,^25−28^ resulting in impaired gut barrier function. DSS administration led to significant downregulation of gut barrier-associated proteins, while MUC2, ZO-1, occludin, and claudin-4 levels were significantly higher in the BC198 group than in the DSS group (Figure 4A–F). In addition, occludin, claudin-4, and E-cadherin levels were higher in mice treated with heat-killed *B. coagulans* BC198 than in the DSS group (Figure 4A–H). These results suggest that both live and heat-killed *B. coagulans* BC198 might help reinforce the gut barrier.

The IgA concentration in feces, which increases under the conditions of barrier function and bacterial translocation in IBD patients, was subsequently tested. The IgA concentration of the DSS group showed an 8-fold increase compared to that of the control group (*p* < 0.001) (Figure 4K); in contrast, the IgA concentration of the BC198 and HKBC198 groups was reduced to half that of the DSS group (*p* < 0.05). The results for gut barrier-associated protein levels and IgA concentration suggest that supplementation with live or heat-killed *B. coagulans* BC198 enhanced the gut barrier function, thereby reducing the extent of bacterial translocation, which may contribute to the reduction in neutrophil and M1 macrophage infiltration of colon tissue (Figure 3G).

### Live *B. coagulans* BC198 Increased Anti-Inflammatory Tregs in Colon Tissue

**Figure 5:**
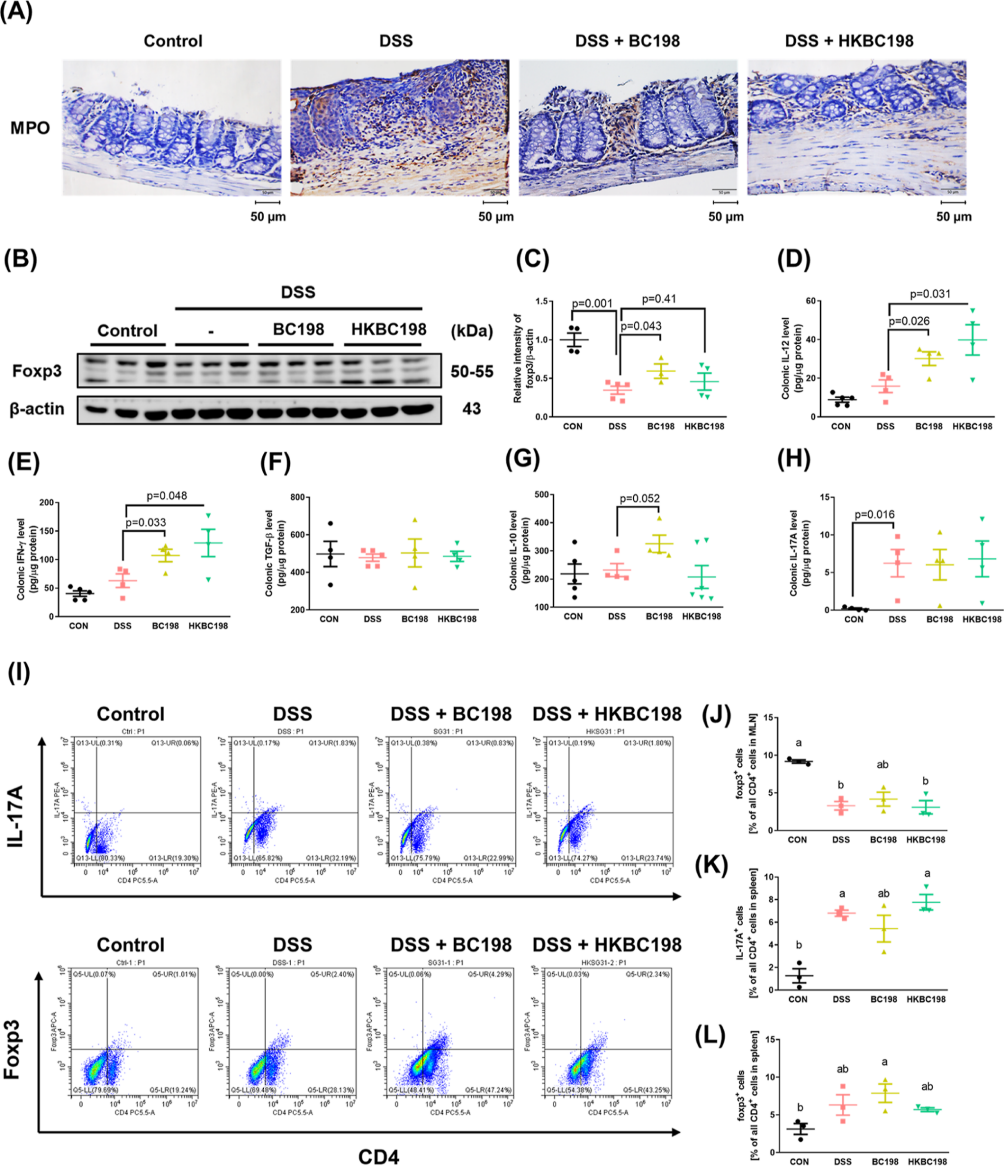
Live *B. coagulans* BC198 corrected the Th17/Treg imbalance caused by colitis. (A) Immunohistochemical staining of MPO protein expression (brown) in distal segments of colon tissue (400× magnification; scale bar = 50 μm); (B,C) Western blot detection and quantification of Foxp3 protein expression in colon tissue. ELISA analysis of (D) IL-12; (E) IFN-γ; Treg-associated anti-inflammatory cytokines (F) TGF-β and (G) IL-10; and (H) Th17-associated cytokine, IL-17A, in the colon supernatant. (I) Flow cytometric analysis of Th17 cells and Treg cells in mouse MLN. (J) Percentage of Foxp3^+^ Treg cells in all CD4^+^ T cells in the mouse MLN. (K) IL-17A^+^ Th17 cells and (L) Foxp3^+^ Treg cells in all CD4^+^ in the mouse spleen. Data are shown as mean ± standard error. Significant differences were analyzed using either two-tailed Student’s *t*-tests or one-way ANOVA followed by Tukey’s HSD posthoc test. The letters (a, b) represent significantly different groups (*p* < 0.05). .

Neutrophil and M1 macrophage infiltration of colon tissue leads to its destruction during inflammation,^29,30^ and the pro-inflammatory cytokines and free radicals produced by these innate immune cells may also cause damage to epithelial cells. Therefore, we examined the effects of live and heat-killed *B. coagulans* BC198 on neutrophil and M1 macrophage infiltration. Immunohistochemical staining of myeloperoxidase (MPO), a biomarker of neutrophils, showed high expression and distribution of MPO in the mucosa and submucosa of colon tissue of the DSS group; in the BC198 and HKBC198 groups, MPO expression was observably lower than that in the DSS group, and the protein was only distributed in the mucosa, indicating that supplementation with live or heat-killed *B. coagulans* BC198 reduced neutrophil infiltration (Figure 5A).

To explore the effects of *B. coagulans* BC198 on the Th17-Treg balance, which is crucial to immune homeostasis, changes in Th17 and Treg cells in the spleen of mice were examined using flow cytometry. The proportion of Th17 cells was significantly higher in the DSS group than in the control group (*p* < 0.05) (Figure 5K), indicating that there was an imbalance in the normal Th17/Treg ratio, which also occurs in IBD patients and other animal models of colitis.^31−34^ However, the percentage of Treg cells in the BC198 group was higher (Figure 5), indicating that supplementation with live *B. coagulans* BC198 restored the balance between pro-inflammatory and anti-inflammatory responses.

In addition, IL-17A—a cytokine produced by Th17 cells—IL-10, and TGF-β—a cytokine produced by Treg cells in the colon—were also detected by ELISA analysis. Results showed that although the IL-17A concentration was not reduced in the BC198 group compared to the DSS group (Figure 5H), the IL-10 concentration in the BC198 group tended to increase (Figure 5G), while none of the cytokines showed differences in concentration between the HKBC198 group and the DSS group (Figure 5F–H). To confirm whether the increase in IL-10 concentration in the BC198 group was related to an increase in Treg cells, Foxp3 protein expression in the colon was examined with Western blotting. The amount of Foxp3 protein was significantly higher in the BC198 group than in the DSS group (*p* < 0.05), while there was no significant difference between the HKBC198 group and the DSS group (Figure 5C). These results confirmed that supplementation with live *B. coagulans* BC198 could enhance Treg cells and the anti-inflammatory response in the colon. However, no positive effect was observed in the HKBC198 group, which may explain the greater effectiveness of the live form in ameliorating colitis compared with the heat-killed form.

### Live and Heat-Killed *B. coagulans* BC198 Corrected the Gut Microbial Dysbiosis Induced by Colitis

**Figure 6:**
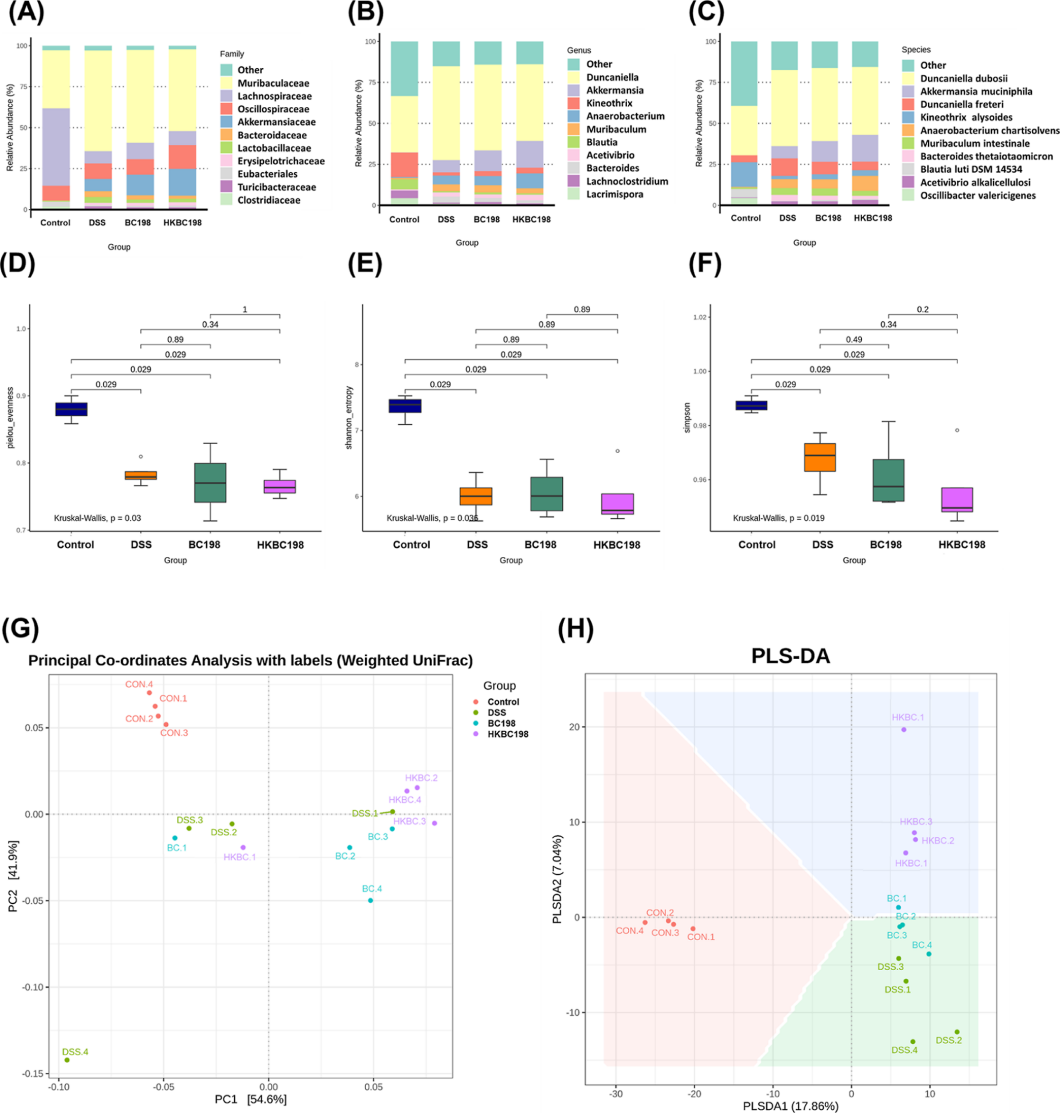
Effects of live and heat-killed *B. coagulans* BC198 on gut microbiota composition. The top 10 relative abundances by taxon rank of the (A) family; (B) genus; and (C) species of the groups compared. Alpha diversity indices: (D) Pielou’s evenness; (E) Shannon index; and (F) Simpson index. Beta diversity indices: (G) PCoA (weighted uniFrac); and (H) PLS-DA.

**Figure 7:**
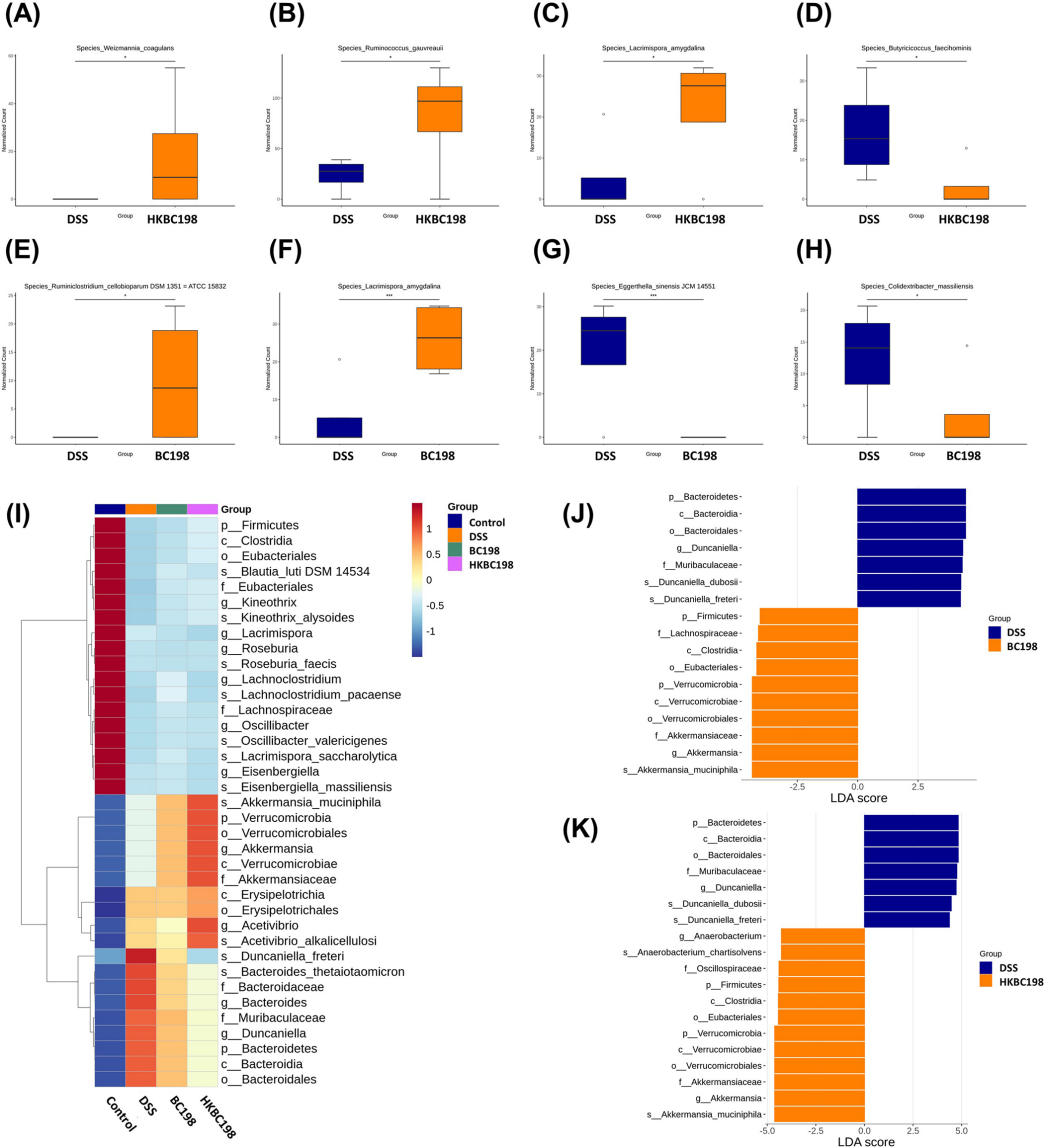
Biomarker identification for each group. Species showing significant differences between (A–D) DSS and HKBC198 and (E–H) DSS and BC198 were identified by metagenomic sequencing. Linear discriminant analysis effect size (LEfSe) of microbial biomarkers of each group, which represent species significantly more enriched in each group compared to other groups with an LDA score >4. The colors blue, orange, green, and purple represent the control, DSS, BC198, and HKBC198 groups, respectively. (I) Heatmap shows the relative abundance of biomarkers in each sample from the four groups. (J) LEfSe analysis of discriminative species compared between DSS (blue) and BC198 (orange) groups. (K) LEfSe analysis of discriminative species compared between DSS (blue) and HKBC198 (orange) groups.

The top 10 relative abundances of gut bacteria at the taxon levels of family, genus, and species are presented in Figure 6A–C. DSS treatment observably changed the gut microbial composition, while supplementation with live BC198 or heat-killed BC198 led to some modulatory effects, for instance, an increase in *Akkermansia muciniphila* and a reduction in *Duncaniella freteri* (Figure 6A–C). In contrast, DSS treatment significantly reduced gut microbiota evenness, as determined by Pielou’s evenness index, Shannon entropy, and the Simpson index (Figure 6D–F). However, neither live BC198 supplementation nor heat-killed BC198 supplementation reversed the decrement. Therefore, β-diversity indices were determined to confirm the similarity in the gut microbial composition (Figure 6G–H). Supplementation with live or heat-killed *B. coagulans* BC198 led to compositional changes. As depicted in Figure 6G,H, the CON group is noticeably separated from the other experimental groups, indicating a comparatively distinct composition of gut microbiota in the CON group. In comparison, HKBC could have a greater effect on compositional changes than BC, as reflected by the shifting distance (Figure 6H), and this result is supported by Figure 7I, which shows that HKBC could facilitate the growth of *Akkermansia* and *Acetivibrio* more than BC did. However, the underlying reason for this needs to be clarified in the future.

Linear discriminant analysis effect size (LEfSe) was employed to determine the biomarkers of each group (Figure 7I–J). The results showed that both BC198 and HKBC198 facilitated similar bacterial growth, e.g., by promoting *A. muciniphila* and *Acetivibrio alkalicellulosi* and inhibiting *D. freteri* and *Bacteroides thetaiotaomicron* (Figure 7I). Notably, HKBC198 had a more influential effect on gut microbiota modulation than BC198 (Figure 7I). *Duncaniella dubosii* showed a significant increase in the DSS group compared to the BC198 or HKBC198 group (Figure 7J–K). *A. muciniphila* and *A. alkalicellulosi* were found to facilitate the growth of each other (Figure S2), indicating that both *B. coagulans* treatments could lead to the formation of a gut microbial guild.

The biomarker of the HKBC198 group was *A. muciniphila* (Figure 7I–K). Many studies have shown that *A. muciniphila* is an important commensal microbe that promotes gut barrier function and is particularly associated with the quality of the mucus layer and the expression of tight junction proteins.^35−37^*A. muciniphila* abundance was found to be significantly lower in the mucosa and fecal samples from patients with ulcerative colitis.^38,39^ The abundance of *A. muciniphila* was higher in both the live and the heat-killed *B. coagulans* BC198 groups than in the DSS group. This suggests that both forms of *B. coagulans* BC198 were beneficial to *A. muciniphila* growth, which may further promote MUC2 and tight junction protein expression.

It is noteworthy that the BC198 group, as well as the HKBC198 group, showed higher abundances of Firmicutes, Clostridia, Eubacteriaceae, and Lachnospiraceae (Figure 7), indicating that both live and heat-killed *B. coagulans* BC198 improved the dysbiosis induced by colitis. Furthermore, metagenomic sequencing showed that HKBC198 facilitated the growth of *Weizmannia coagulans* and *Ruminococcus gauvreauiiDSM1351* and inhibited *Butyricicoccus faecihominis* (Figure 7A–D), while BC198 induced the growth of *Ruminiclostridium cellobioparum* and inhibited *Eggerthella sinensisJCM14551* and *Colidextribacter massiliensis*. Notably, both *B. coagulans* treatments could enhance the growth of *Lacrimispora amygdalina*.

## Discussion

The present study showed that the live form of *B. coagulans* BC198 ameliorated the severity of colitis (Figure 1B), prevented colon shortening (Figure 1F), and reduced colitis-induced inflammation and damage to colon tissue (Figure 2E,F) by reducing neutrophil and M1 macrophage infiltration of colon tissue (Figure 3), possibly by enhancing the gut barrier function (Figure 4) and increasing the levels of Treg cells and the anti-inflammatory cytokine, IL-10, in the colon (Figure 5). These mechanisms of action may also be related to the increase in beneficial commensals and the correction of gut microbial dysbiosis (Figure 6). In contrast, although the heat-killed form of *B. coagulans* BC198 also improved the gut barrier function (Figure 4) and reduced neutrophil and M1 macrophage infiltration (Figure 3), it was unable to increase the level of Treg cells and IL-10 (Figure 5), which may account for its lower effectiveness in ameliorating the severity of colitis (Figure 1).

The effectiveness of *B. coagulans* BC198 in improving DSS-induced colitis was evaluated using the DAI (Figure 2E), the shortening of the colon (Figure 1), histological parameters (Figure 2G), and pro-inflammatory cytokines in colon tissue (Figure 3A–D). All results indicated that the live form was more effective than the heat-killed form in ameliorating colitis. The reasons for the difference between live and heat-killed *B. coagulans* BC198 were investigated by examining their effects on gut barrier function, innate and adaptive immunity, and gut microbiota.

In the present study, the live and heat-killed *B. coagulans* BC198 treatments showed an increase in the expression of gut barrier-associated proteins (Figure 4), MUC2, and tight junction proteins, which are reduced in patients with IBD.^25−28^ Lipoteichoic acid (LTA), embedded in the cell wall of Gram-positive bacteria, is a ligand for pattern recognition receptors (PRR), such as the platelet-activating factor receptor (PAFR) and toll-like receptor 2 (TLR2) of intestinal epithelial cells. The combination of LTA and PAFR promotes the production of MUC2 protein,^40,41^ and the combination of LTA and TLR2 promotes the expression of tight junction proteins.^42−48^ This suggests that live and heat-killed *B. coagulans* BC198 promoted the expression of gut barrier-associated protein via LTA or other cell wall components of intestinal epithelial cells that bind PRR to induce related reactions. The increased expression of gut barrier-associated proteins in the BC198 and HKBC198 groups could indeed enhance gut barrier function, as verified by the decrease in IgA compared to the DSS group (Figure 4K), since increased IgA concentrations have been observed in other studies under conditions of gut barrier defects, increased intestinal permeability, and bacterial translocation.^49,50^ An increase in IgA has also been found in Crohn’s Disease and ulcerative colitis patients, and there was a positive correlation between IgA and the blood C-reactive protein, a marker of inflammation, and the DAI, the Mayo score.^51^ Since impaired gut barrier function leads to the translocation of luminal bacteria to the lamina propria of the gut mucosa, thereby activating immune cells in the lamina propria and attracting monocytes and neutrophils from the blood to the colon.^24^ It is possible that the reduction in neutrophils and M1 macrophage infiltration caused by live and heat-killed *B. coagulans* BC198 (Figure 3) was mediated through enhancement of the gut barrier function (Figure 4).

In the adaptive immune response, the Th17:Treg cell ratio determines whether the inflammatory disease can be alleviated.^8^ Supplementation with live *B. coagulans* BC198 increased Treg cells and the anti-inflammatory response in the colon, while the heat-killed form did not have this effect (Figures 3 and 5), which may be the reason for the greater effectiveness of the live form than the heat-killed form in ameliorating colitis (Figure 1). *Bacillus* spp. produce acetic acid after fermentation of carbohydrates;^52,53^ therefore, the increase in acetic acid may be due to live *B. coagulans* BC198, while the heat-killed form cannot produce such metabolites and, therefore, does not promote Treg cell accumulation in the colon. Acetic acid is a ligand for G protein-coupled receptor 43 (GPR43) on the surface of immune cells, whose activation promotes the homing of Treg cells from the peripheral bloodstream to the colon and also promotes the accumulation of Treg cells in the colon.^54,55^ The severity of DSS-induced colitis is reduced in mice fed with acetic acid,^56^ suggesting that acetic acid may indeed have an anti-inflammatory effect by increasing Treg cells in the colon.

Dysbiosis in IBD may be responsible for impaired gut barrier function and a dysregulated immune response.^24^ Both the live and the heat-killed form of *B. coagulans* BC198 increased the abundance of *A. muciniphila* (Figure 7), a species that promotes the quality of the mucus layer and the expression of tight junction proteins,^35−37^ which might have contributed to the improvement in the gut barrier function seen in the BC198 and HKBC198 groups (Figure 4).

According to one of the latest reviews of *B. coagulans*, several benefits can be attributed to different *B. coagulans* strains, including the production of enzymes, regulation of imbalanced microbiota, maintenance of human immunologic homeostasis, and treatment of various gastrointestinal diseases.^11^ In our study, we observed that live BC198 exhibited a more significant effect on balancing immunologic homeostasis compared to that of heat-killed BC198. This could be attributed to the potential benefits to the immune system, which may be linked to gut microbial metabolites or their enzymes. Similar findings have been reported in previous studies, suggesting a more pronounced effect of live probiotics compared to their heat-killed counterparts, owing to the regulation of intestinal metabolism.^57^ Therefore, our study suggests that live *B. coagulans* BC198 may have a better effect on ameliorating IBD than its heat-killed counterpart.

## Future Perspectives

Developing probiotics into regenerative medicine for IBD presents some challenges. According to this definition, probiotics can be considered live biotherapeutic products. In our study, BC198 was found to exert a modulatory effect on the immune response. Recently, the transplantation of stem cells to restore microcirculation and accelerate the repair of the intestinal epithelium has emerged as a major idea. It is worth mentioning that the immunomodulatory effect of hematopoietic stem cells might play a crucial role in reducing the inflammatory responses of IBD patients. Attenuating immune-mediated organismal damage could contribute to the immunomodulatory functions therein.^58^ Similar to other developed live biotherapeutic products, immunomodulation is a major potential mechanism.^59^ However, there are still challenges and approaches to overcome in developing BC198 as a regenerative medicine. For instance, more preclinical results should be confirmed.

## Conclusions

The results of this study show that the live form of *B. coagulans* BC198 is more effective in ameliorating colitis than the heat-killed form. Live *B. coagulans* BC198 ameliorated colitis by increasing gut barrier-associated protein expression, reducing neutrophil and M1 macrophage infiltration of colon tissue, decreasing the Th17/Treg ratio in the spleen, and increasing Treg cells and their anti-inflammatory cytokine, IL-10, in the colon. These beneficial effects may be related to the role of *B. coagulans* in regulating the gut microbiota, including increasing the relative abundance of beneficial commensal species that promote Treg cell accumulation in the colon. In contrast, the heat-killed form failed to enhance the anti-inflammatory response in the colon, which might have resulted in its lack of the colitis-ameliorating effects shown by the live form.

## Materials and Methods

### Live and Heat-Killed *B. coagulans* Strain

The live bacterial samples consisted of a freeze-dried powder of the selected probiotic strain *B. coagulans* BC198, isolated from green malt. The heat-inactivated bacterial samples consisted of freeze-dried powder of the inactivated bacteria after heating at 70 °C for 30 min. The freeze-dried and heat-killed probiotic powders were provided by Syngen Biotech Co., Ltd. (Taiwan).

### DSS-Induced Colitis Model and Treatments

The experimental animals were four-week-old male C57BL/6 mice purchased from BioLASCO Taiwan Co., Ltd. The animals were housed at 25 ± 2 °C and 40–60% relative humidity under 12 h of light and 12 h of darkness. The animals were given sufficient water and food. The mice were randomly assigned to one control group and three induction groups according to the average body weight of each cage after 2 weeks of prerearing. The drinking water of the three induction groups was changed to an aqueous solution containing 3% DSS to induce colitis until day 6 of the experiment. Then, these mice were randomly assigned to the DSS group and the *B. coagulans* treatment groups, BC198 for the live probiotic and HKBC198 for the heat-killed probiotic, according to their average body weight after induction of colitis (day 6). The drinking water of all three groups was changed to normal pure water on day 6 to simulate a period of recovery from colitis, and tube feeding with *B. coagulans* began: each mouse in the BC198 group was tube-fed with 0.15 mL of a 20% *B. coagulans* BC198 solution containing 1 × 10^9^ cfu of live *B. coagulans* BC198, and each mouse in the HKBC198 group was tube-fed with 0.1 mL of a 20% *B. coagulans* HKBC198 solution containing 1 × 10^9^ cells of heat-killed *B. coagulans* BC198. Each mouse in the control group (which did not receive DSS) and the DSS group was administered a phosphate-buffered saline (PBS) solution once a day until the end of the experiment. On day 20, the drinking water of the BC198, HKBC198, and DSS groups was changed to an aqueous solution containing 2% DSS for 5 days to simulate the recurrence of colitis. On day 25, the drinking water was changed back to normal pure water until the end of the experiment on day 27 to simulate a period of recovery from colitis. The experimental design is shown in Figure S1.

### Evaluation of the DAI

The DAI—the average weight loss score, stool consistency score, and rectal bleeding score—was recorded every 2 days. The weight-loss score was as follows: 0 = weight loss <1% of the initial weight; 1 = 1–5%; 2 = 5–10%; 3 = 10–15%; and 4 = >15%. The stool consistency score was 0 = well-formed pellets; 1 = soft pellets not adhering to the anus; 2 = very soft pellets adhering to the anus; 3 = liquid stool in long streams and wet anus; and 4 = diarrhea. The rectal bleeding score was 0 = hemoccult negative; 1 = hemoccult positive (light green); 2 = hemoccult positive (light blue); 3 = hemoccult positive (dark blue); and 4 = gross bleeding.^21^

### Histological Examination of Colon Tissue

The histological score of colon tissue was determined based on the sum of inflammation severity (0 = none, 1 = mild, 2 = moderate, and 3 = severe), inflammation extent (0 = none, 1 = mucosa, 2 = submucosa, and 3 = transmural), crypt damage (0 = none, 1 = basal 1/2 damage, 2 = basal 2/3 damage, 3 = crypt lost but surface epithelium present, and 4 = crypt and surface epithelium lost), and percent involvement (0 = 0%, 1 = 1–25%, 2 = 26–50%, 3 = 51–75%, and 4 = 76–100%).^60^

### Cytokine Assay of the Supernatant of the Colon Biopsies Cultured Ex Vivo

The colon was then washed with PBS containing antibiotics, immersed in a 24-well dish containing RPMI1640, and incubated for 24 h at 37 °C in an incubator. All cultures were then aspirated into a microcentrifuge tube and centrifuged at 12,000 rpm for 1 h. The supernatant was used to conduct cytokine assays with an ELISA kit (Invitrogen, CA, US).

### Immunohistochemical Staining for MPO

For immunohistochemical staining, sections of paraffin-embedded tissue were stained with MPO antibody (1:500), goat antirabbit HRP-conjugate, and hematoxylin (Leica, Wetzlar, Germany).

### Immunofluorescence Staining for iNOS

For immunofluorescence staining, sections of paraffin-embedded tissue were stained with iNOS antibody (Abcam), goat antirabbit HRP-conjugate, and DAPI. For the fluorescence analysis of the images, the average immunofluorescence value was calculated with all images from one sample. Computerized quantification of the mean fluorescence intensity and coverage rate of images was analyzed by using ImageJ software.

### Western Blot Analysis

Colon tissue was weighed and homogenized in a cold lysis buffer. The homogenate was then centrifuged at 4 °C for 30 min at 12,000 rpm, and the supernatant was collected. MUC2, ZO-1, occludin, claudin-4, E-cadherin, and Foxp3 protein expression in the supernatant of the colon tissue homogenate was measured by using Western blot. Protein samples were separated in 8–12% SDS-PAGE and then transferred to PVDF membranes. The membranes were blocked with blocking solution for 2 h and then reacted with primary antibodies against MUC2 (Santa Cruz, Texas, US), E-cadherin, and ZO-1 (Cell Signaling, MA, US), occluding claudin-4 and Foxp3 (Proteintech Inc., IL, US), and β-actin (Sigma-Aldrich, MA, US) as an internal control at 4 °C overnight. Membranes were then incubated with the second antibodies at room temperature for 1 h, and bands were visualized using ECL. The intensities of the bands were measured using ImageJ software.

### T-Cell Extraction from the Spleen

#### Preliminary Disassembly of Connective Tissue from the Spleen

The spleen was removed from the mouse and placed in a collagenase solution (1 mg/mL) for 15 min.

#### Extraction of Cells

The spleen and collagenase solution were placed in a dish, and the organ was pressed with the tip of a 3 mL syringe for 2 min to initially disintegrate the tissue. The solution was then filtered through a 70 μm cell filter system (a 50 mL centrifuge tube was placed underneath the filter to receive the filtered solution), and the head of the syringe was used to grind the initially disintegrated visceral tissue on the filter until no tissue fragments remained. Finally, 5 mL of PBS was used to wash off the material adhering to the cell filter mesh in the lower centrifuge tube. After the supernatant was removed, 30 mL of PBS harvesting medium was added to dissolve the cell precipitate, which was then centrifuged again. After removing the supernatant, 1 mL of the harvesting medium was added to resolubilize the cellular precipitate.

#### Lysis of Erythrocytes

5 mL of lysing buffer was added to the above-mentioned centrifuge tube containing the cell solution; the tube was gently shaken to allow the lysing buffer to act fully and placed in an incubator at 37 °C for 10 min, protected from light. The supernatant was carefully aspirated; 2 mL of stain buffer was added to it to wash off the lysis buffer, and it was then centrifuged at 200*g* for 5 min at 4 °C. The cell precipitate was then solubilized with 1 mL of stain buffer. The cells were aspirated into a 24-well dish and placed in an incubator until further processing.

### Th17/Treg Immunophenotyping

#### Activation of Th17 Cells

5 μL of 10 μg/mL PMA and 5 μL of 100 ng/mL Ionomycin were added to each well of a 24-well dish with 1 mL of spleen or MLN cytosol per well to activate Th17 cells to produce IL-17. After 1 h of reaction in the incubator, 5 μL of GolgiStop (BD Biosciences, cat. no. 560767) was added to prevent Th17 cells from releasing IL-17 outside the cells, and the cells were left in the incubator for another 5 h. The cells from the well plate were centrifuged in a 1.5 mL microcentrifuge tube at 300*g* for 5 min to remove the effector solution. The supernatant was removed after centrifugation, and the cells were lysed with 1 mL of stain buffer (BD Biosciences, Cat no. 554656). The cells were then counted.

#### Cell Fixation

After the supernatant was removed by centrifugation at 300*g* for 5 min, the cell precipitate was gently redissolved with the remaining stain buffer. Then, 200 μL of Foxp3 fixation buffer was added and mixed well, and the cells were allowed to react at 4 °C for 30 min.

#### Permeabilization

After centrifuging the supernatant at 500*g* for 5 min, 200 μL of prewarmed (37 °C) Foxp3 permeabilization buffer was added to the cell precipitate to gently resolubilize it. The reaction reagent was then removed by centrifugation at 500*g* for 5 min, and 1 mL of stain buffer was added to the cell precipitate to redissolve it and wash off the residual reaction reagent. The stain buffer was removed by centrifugation at 500*g* for 5 min, and the washing procedure was repeated once more.

#### Cell Staining

250 μL of cytosol was divided into five 1.5 mL microcentrifuge tubes of 50 μL each. One tube was without any reagent, and the other four tubes contained 5 μL of PerCP-Cy 5.5 rat antimouse CD4 reagent (BD Biosciences, Cat no. 561115), 5 μL of PE rat antimouse IL-17A (BD Biosciences, Cat no. 561115), and 5 μL of PE rat antimouse IL-17A (BD Biosciences, Cat no. 561115), mouse IL-17A (BD Biosciences, Cat no. 561020), Alexa Fluor 647 rat antimouse Foxp3, and Th17/Treg phenotyping cocktail (BD Biosciences, Cat no. 560767). The reaction was carried out for 30 min at room temperature, protected from light. After the reaction was completed, 1 mL of stain buffer was added, and the solution was centrifuged at 500*g* for 5 min to remove the stain, and the washing procedure was repeated once more. Finally, the cell precipitate was dissolved in 500 μL of stain buffer, and it was ready for use.

### Statistical Analysis

Data are expressed as the mean ± standard error. Statistical analysis (ANOVA or Student’s *t*-test) was performed using SPSS. Differences were considered significant at *p* < 0.05.

## Supporting Information Available

Experimental design and network of correlations between species identified in this study (PDF)
