# Supplementary material for: Efficacy and Mechanism of the Action of Live and Heat-Killed Bacillus coagulans BC198 as Potential Probiotic in Ameliorating Dextran Sulfate Sodium-Induced Colitis in Mice
Source: ACS Omega. 2024 Feb 20;9(9):10253–66. doi: 10.1021/acsomega.3c07529 (PMC10918820; doi:10.1021/acsomega.3c07529)
Supplement: Supplementary file 1 — ao3c07529_si_001.pdf [file ao3c07529_si_001.pdf]

Supporting materials

Efficacy and mechanism of action of live and heat-killed *Bacillus coagulans* BC198 as potential probiotic in ameliorating DSS-induced colitis in mice

Yen-Chun Koh[1#], Ya-Chu Chang[1#], Wei-Sheng Lin[1,2], Siu-Yi Leung[1], Wei-Jen Chen[3], Shiuan-Huei Wu[3], Yu-Shan Wei[4], Chiau-Ling Gung[4], Ya-Chun Chou[1], Min-Hsiung Pan[1,5,6]

[1] Institute of Food Sciences and Technology, National Taiwan University, Taipei, Taiwan
[2] Department of Food Science, National Quemoy University, Quemoy, Taiwan
[3] Biotech Department, Syngen Biotech Co., Ltd., Tainan, Taiwan
[4] Research and Development Department, Syngen Biotech Co., Ltd., Tainan, Taiwan
[5] Department of Medical Research, China Medical University Hospital, China Medical University, Taichung City, Taiwan
[6] Department of Health and Nutrition Biotechnology, Asia University, Taichung City, Taiwan

# The authors contribute equally
* Please send all correspondence to:
Dr. Min-Hsiung Pan
Institute of Food Science and Technology,
National Taiwan University,
No. 1, Section 4, Roosevelt Road, Taipei 10617, Taiwan.
Tel. no. +886-2-33664133
Fax. no. +886-2-33661771
E-mail: mhpan@ntu.edu.tw

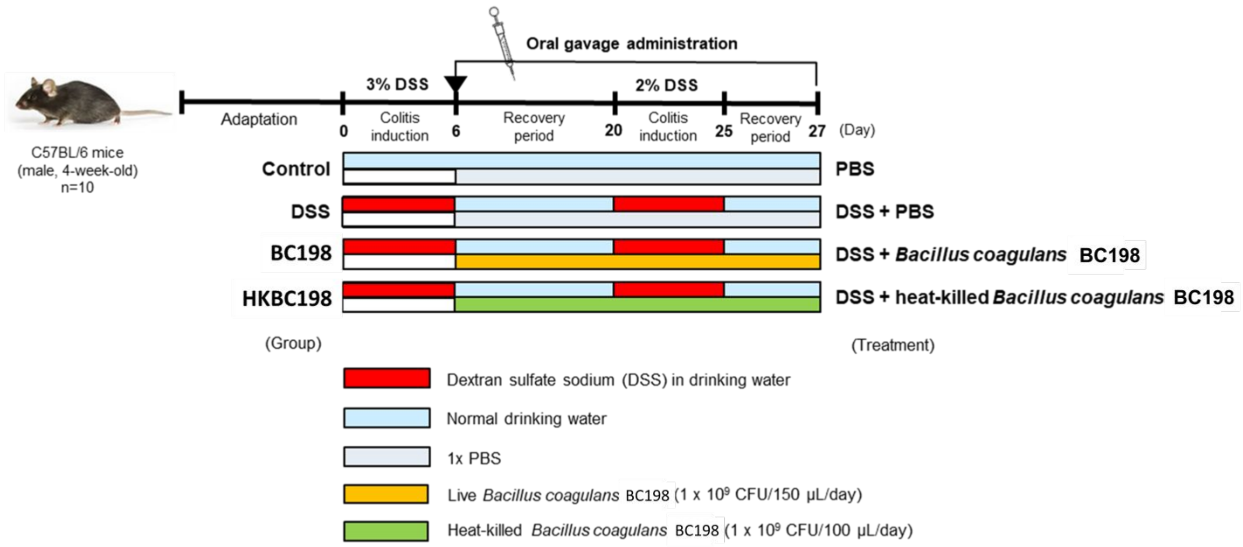

Figure S1. Experimental design.

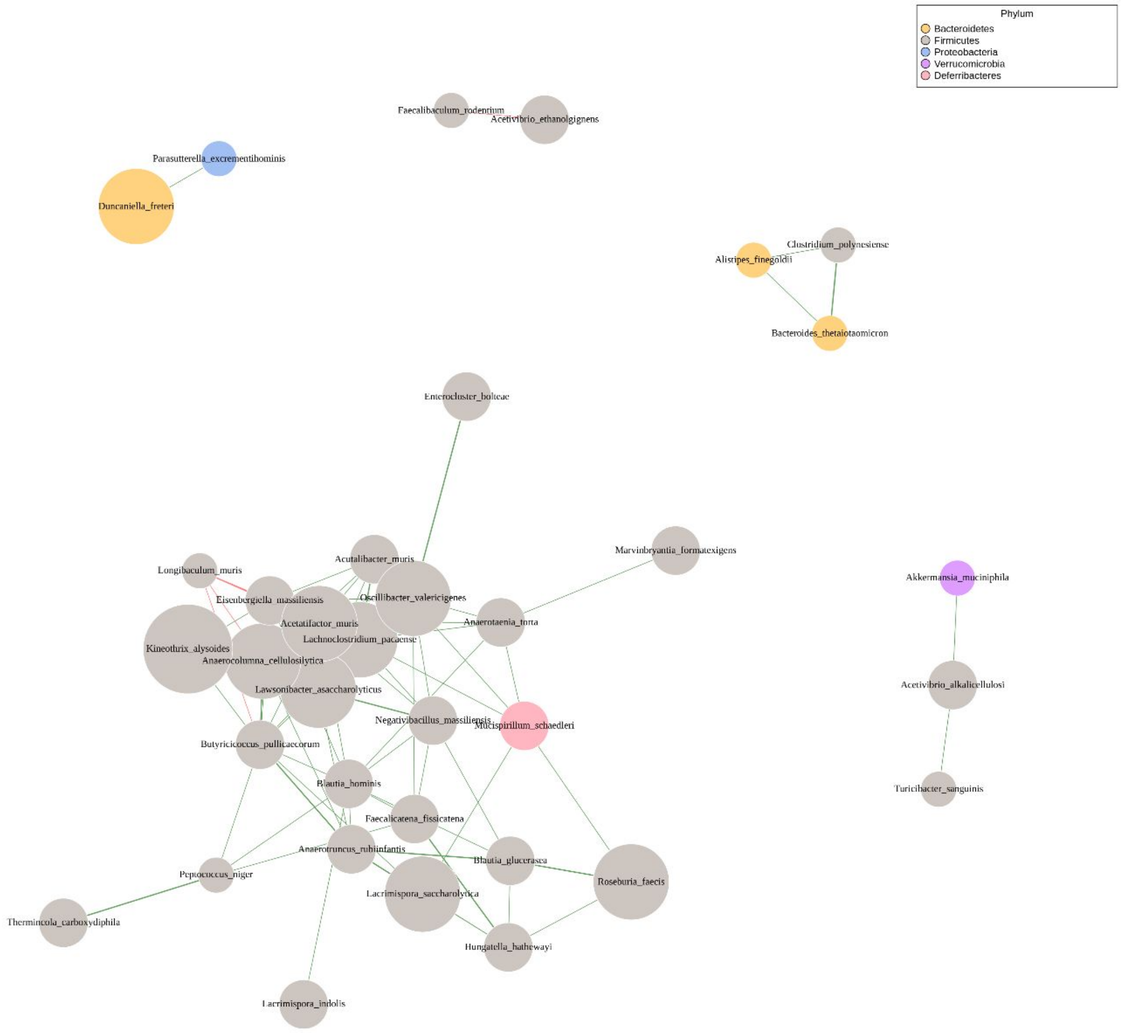

Figure S2. Network of correlations between species identified in this study.

The threshold correlation coefficient was set at 0.8. The species presented above were significantly different between all experimental groups.
